# Individual preferences for certain types of spirals and the possible role of creativity

**DOI:** 10.1038/s41598-025-14326-z

**Published:** 2025-08-06

**Authors:** Ronald Hübner, Golfam Goodarzi

**Affiliations:** 1 Department of Psychology, University of Konstanz, Konstanz, Germany; 2 Department of Psychology, University of Konstanz, Konstanz, Germany

**Keywords:** Archimedean spiral, Golden spiral, Aesthetic preference, Individual differences, Creativity, Psychology, Human behaviour

## Abstract

This study explores the appeal of different types of spirals, commonly found in nature, architecture, and art. The first experiment investigated aesthetic appreciation of Archimedean, logarithmic, and golden spirals, considering factors such as size and the number of turns. As a result, three groups of individuals were identified: those who favored golden spirals, those who preferred Archimedean spirals, and a group that liked logarithmic spirals and similar ones. Interestingly, personality traits showed no clear relation with these preferences. In the first part of a second experiment, the participants were not only asked to choose between different types of spirals, but also to justify their choice by providing reasons. The results indicate that the preferences often stem from the spirals’ features as well as from personal associations, confirming that aesthetic judgments are influenced by both objective and subjective factors. However, results also suggested that the preference for certain types of spirals is related to creativity. This hypothesis was tested in the second part of the second experiment in which participants generated associations to the spirals. The analysis of the frequency and originality of the associations revealed that individuals who prefer the golden spiral to the Archimedean spiral tend to be more creative. Overall, the study highlights the complexity of aesthetic preferences and the potential role of both objective features and subjective associations in shaping our responses to spirals.

## Introduction

Spirals, a recurring motif in nature, architecture, art, etc., have exerted a great attraction on people for millennia^1^. In his 1914 book “The Curves of Life”, the British art critic and writer Theodore Andrea Cook (1867–1928) examined various aspects of spirals and presented an impressive 426 examples, ranging from plant and shell structures to the human body and the Andromeda Nebula^2^. He also examined their beauty and speculated on our reaction to them. Cook assumed that spirals are associated with growth, a fundamental principle of life, and that this is an important source of their beauty.

Although spirals have become somewhat less important as ornaments in some areas such as architecture and furniture design, they are still a universally popular theme^3,4^. Unfortunately, the question of why we find them beautiful is still largely unanswered. One might think that our appreciation of spirals is related to their common occurrence in nature, or that the beauty of spirals lies in their mathematical properties that evoke a sense of harmony. However, convincing empirical evidence for such ideas is rare.

Furthermore, spirals can be categorized into different types, e.g., Archimedean spirals, logarithmic spirals, Fibonacci spirals or golden spirals, with each type having unique characteristics. Are these types all equally well liked? Hübner^5^ recently demonstrated that this is not generally the case. In his study, about 80% preferred the golden spiral to the Fibonacci spiral. This high agreement is remarkable, especially, because the two spiral types are very similar. However, they differ in a hardly visible but fundamental characteristic. Whereas the curvature of the golden spiral changes continuously along its path, that of the Fibonacci spiral, which is composed of quarter circles, remains constant within each quarter circle and changes abruptly from one quarter circle to the next. Such abrupt changes in curvature seem to be universally disliked.

In this study, we wanted to investigate beauty differences within spiral types with a continuously changing curvature and effects of size and the number of turns. It is likely that they also differ in their perceived beauty. For instance, as certain types of spirals occur more often in our environment than others, this can affect, due to the mere exposure effect^6^ their aesthetic appreciation. In a pilot study, we indeed found that some spiral types were liked more than others. However, to our surprise and in contrast to the study by Hübner^5^ there were substantial individual differences. Although studies show that people can differ in their preferences for symmetry^7^ or curvature^8,9^ nothing is yet known about individual differences in preference for specific spiral types. Accordingly, we have not only investigated the extent to which the beauty of different spiral types varies, but also corresponding individual differences and their possible origin. However, before reporting the details of our study, we will provide a brief introduction to the types of spirals examined.

### Archimedean spiral

The Archimedean spiral is a relatively simple spiral that has a constant (linear) expansion, i.e., the distance between successive turns increases by a constant amount for each full rotation. Formally, its distance ρ (rho) from the center to one point on the spiral is proportional to the angle *Φ* (PHI) of rotation in radians. However, there is also a generalized version of the Archimedean spiral, which also enables a non-linear expansion. This means that the distance from the center can inflate^10^. The equation of this generalized Archimedean (G-Archimedean) spiral in the polar coordinate system is:

$$\:{\uprho\:}=a{\phi\:}^{\raisebox{1ex}{$1$}\!\left/\:\!\raisebox{-1ex}{$c$}\right.}$$

**Fig. 1:**
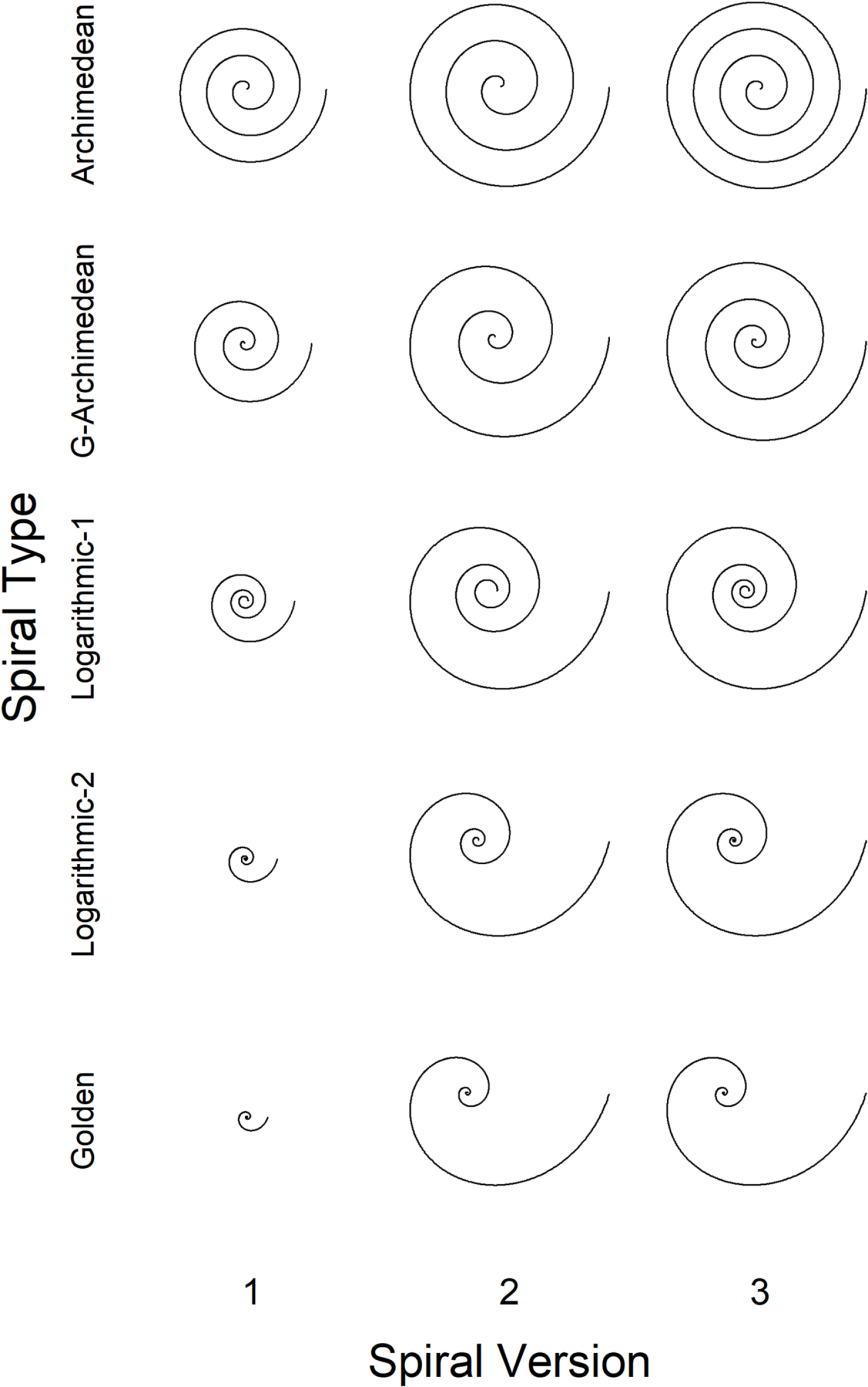
Different types of spirals used as stimuli in our experiments. The versions vary from Version 1 to Version 2 in size, and from Version 2 to Version 3 in number of turns (for details see the text).

While the parameter *a* determines the basic distance between the turns, *c* controls how much it inflates. The standard Archimedean spiral is given if *c* = 1. Examples of Archimedean and generalized Archimedean spirals can be seen in Fig. 1.

### Logarithmic spiral

A logarithmic spiral, equiangular spiral, or growth spiral is a self-similar spiral curve that often appears in nature. The first known description of a logarithmic spiral was provided by Albrecht Dürer^11^ who called it an “eternal line”. More than a century later, it was discussed by Descartes^12^ and then extensively investigated by Jacob I Bernoulli, who called it “spira mirabilis”, i.e., marvelous spiral^10^. The logarithmic spiral differs from the Archimedean spiral in that it expands non-linearly. However, different from the generalized Archimedean spiral, the distance between successive turns increases exponentially. It has the equation:

$$\:r=a{e}^{b\phi\:}$$

The parameter *a* is the polar radius of the spiral point with *Φ* = 0 and thus represents the distance of the origin of the spiral from the polar center. Parameter *b* is often called the *growth* factor. If *b* = 0.3063, then the spiral is a special case, commonly referred to as the *golden* spiral^10^ because it gets wider by a factor that is the golden ratio, for every quarter turn it makes. Although the golden spiral is strictly speaking a logarithmic spiral, we consider it a stand-alone type in this work. Examples of the golden spiral can be seen in Fig. 1.

### The present study

The aim of this study was to investigate the perceived beauty of different types of spirals and the effect of their size and number of turns. Furthermore, we wanted to examine individual differences and their origin. In our pilot study, we had identified three groups of persons that prefer Archimedean, logarithmic, and golden spirals, respectively. However, we had no idea what these different preferences could be related to. In the present study, we therefore investigated whether they are related to personality traits. For this objective, we also administered a short version of the Big Five Inventory (BFI)^13^ to measure the Big Five dimensions of personality: *Openness to experience*, *Conscientiousness*, *Extraversion*, *Agreeableness*, and *Neuroticism*. There are several studies, showing relations between these dimensions and aesthetic preferences. Salimun, et al.^14^ for instance, observed a positive relation between Agreeableness and the preference for aesthetic web pages, while Van Geert and Wagemans^15^ found a negative relation between Openness to experience and preference for visual order. Furthermore, Furnham and Walker^16^ found a positive relation between Neuroticism and preference for abstract and pop art.

Rammstedt and John^17^ developed a ten-item short version (BFI-10) of the BFI. However, since the losses in reliability and validity of the BFI-10 compared to the original BFI were greatest for the Agreeableness scale, Rammstedt and John^17^ recommended adding a specific third item to this scale if the dimension is particularly important. We have followed this advice and will call this eleven-item version “BFI-11”.

After the results of our first experiment not only showed that certain types of spirals are preferred over others, but also that participants differed in this respect, we conducted a second experiment in which we tried to identify possible reasons for the individual choices. Specifically, we directly asked our participants why they prefer the one over the other spiral type. Because the results suggested that associations play an important role, we examined in a short follow-up experiment (Experiment 2B) whether persons differing in preference also differ in specific aspects of creativity.

## Experiment 1

In our first experiment we investigated the extent to which the perceived beauty varies across different types of spirals. Moreover, we also examined effects of size and of the number of turns for each type. For these objectives, we presented each of the spirals shown in Fig. 1 to participants and asked them to rate its beauty. Because we expected individual differences, we also administered the BFI-11 to examine possible relations with personality traits.

### Method

#### Participants

One hundred and eighteen participants (mean age 28.5 years, *SD* = 7.69, 80 cisgender women, 38 cisgender men), most of them students at the University of Konstanz, Germany, were recruited for participation in the experiment. The corresponding online study included not only the present experiment, but also others, which will be reported elsewhere. Participants received a voucher worth EUR 3 for taking part in the study, which lasted around ten minutes in total.

This study was approved by the Institutional Review Board of the University of Konstanz, Germany (approval IRB25KN002-02/w) and conducted in accordance with the ethical guidelines of the University of Konstanz and the Declaration of Helsinki (1964) and its later amendments. Participants were informed of their right to quit the study at any time without reprisal and their informed consent was obtained by check-marking a box before the actual experiment started.

#### Stimuli

Five different types of spirals were used as stimuli: Archimedean (AR), generalized Archimedean (GA), logarithmic-1 (L1), logarithmic-2 (L2), and golden (GO). Each type also varied in the number of turns. As shown in Fig. 1, starting from a 3-turns version (Version 1) of a given spiral type, we increased the number of turns to 4 for obtaining a different version (Version 3). The problem is that increasing the number of turns also increases the spiral’s width. Moreover, as can also be seen in Fig. 1, how much the width increases, depends on the type of spiral. Therefore, to examine the effects of turns and size independently, we additionally created a version (Version 2), which also has 3 turns, but is of the same size as the corresponding 4-turns Version.

Each spiral was first represented in polar coordinates, defined by its radius (*r*) and angle (*Φ*), with *Φ* measured in radians. The plotting started at *Φ* = 0 and incrementally increased by a step size of 0.01 radians up to a final value of *z*π. For each angular step, the corresponding radius and curvature were calculated using their respective equations. The series of polar coordinates was then converted to Cartesian coordinates (*x*, *y*) and plotted as a line graph, generating an image of the spiral. The specific parameters for each spiral type and its versions, along with the corresponding range of *Φ*, are detailed in Table 1.

Our set of spirals allowed us to examine the effect of size on beauty by comparing the ratings between Version 1 and Version 2, and that of the number of turns on beauty by comparing the ratings between Version 2 and Version 3.

**Table 1:** Parameter values of the different spiral types and versions used as stimuli in the experiments. Details on the different parameters can be found in the text above.

Type	Version	Turns	Size	Parameters		
				*a*	*c*	*Range of Φ (PHI)*
Archimedean (AR)	1	3	Normal	0.1315	1	0,…,6π
	2	3	Large	0.1794	1	0,…,6π
	3	4	Normal	0.1315	1	0,…,8π
G-Archimedean (GA)	1	3	Normal	0.0133	0.58	0,…,6π
	2	3	Large	0.0226	0.58	0,…,6π
	3	4	Normal	0.0133	0.58	0,…,8π
Logarithmic-1 (L1)	1	3	Normal	0.1114	0.14	0,…,6π
	2	3	Large	0.2685	0.14	0,…,6π
	3	4	Normal	0.1114	0.14	0,…,8π
Logarithmic-2 (L2)	1	3	Normal	0.014	0. 2262	0,…,6π
	2	3	Large	0.058	0. 2262	0,…,6π
	3	4	Normal	0.014	0. 2262	0,…,8π
Golden (GO)	1	3	Normal	0. 002007	0. 306,349	0,…,6π
	2	3	Large	0. 01378	0. 306,349	0,…,6π
	3	4	Normal	0. 002007	0. 306,349	0,…,8π

#### Procedure

The study began with a short introduction to its topic and procedure. After the participants had given their consent, provided their personal data (gender, age), and completed the BFI-11 questionnaire, specific instructions were given for the main task. To achieve standardized visual quality of stimulus presentation, participants were informed that they had to use a computer. The program stopped if a mobile device was used.

All 15 stimuli were presented in a randomized order for each participant. They appeared in black on a white 500 × 500 pixel square on the screen. However, it should be noted that the exact physical size of the pictures could not be controlled as the screens used probably had different resolutions. The participants’ task was to rate each spiral according to its beauty (from “Not beautiful at all” to “Very beautiful”), on a visual analog scale internally ranging from 1 to 100, where the numbers were not visible.

### Results

#### Ratings

**Fig. 2:**
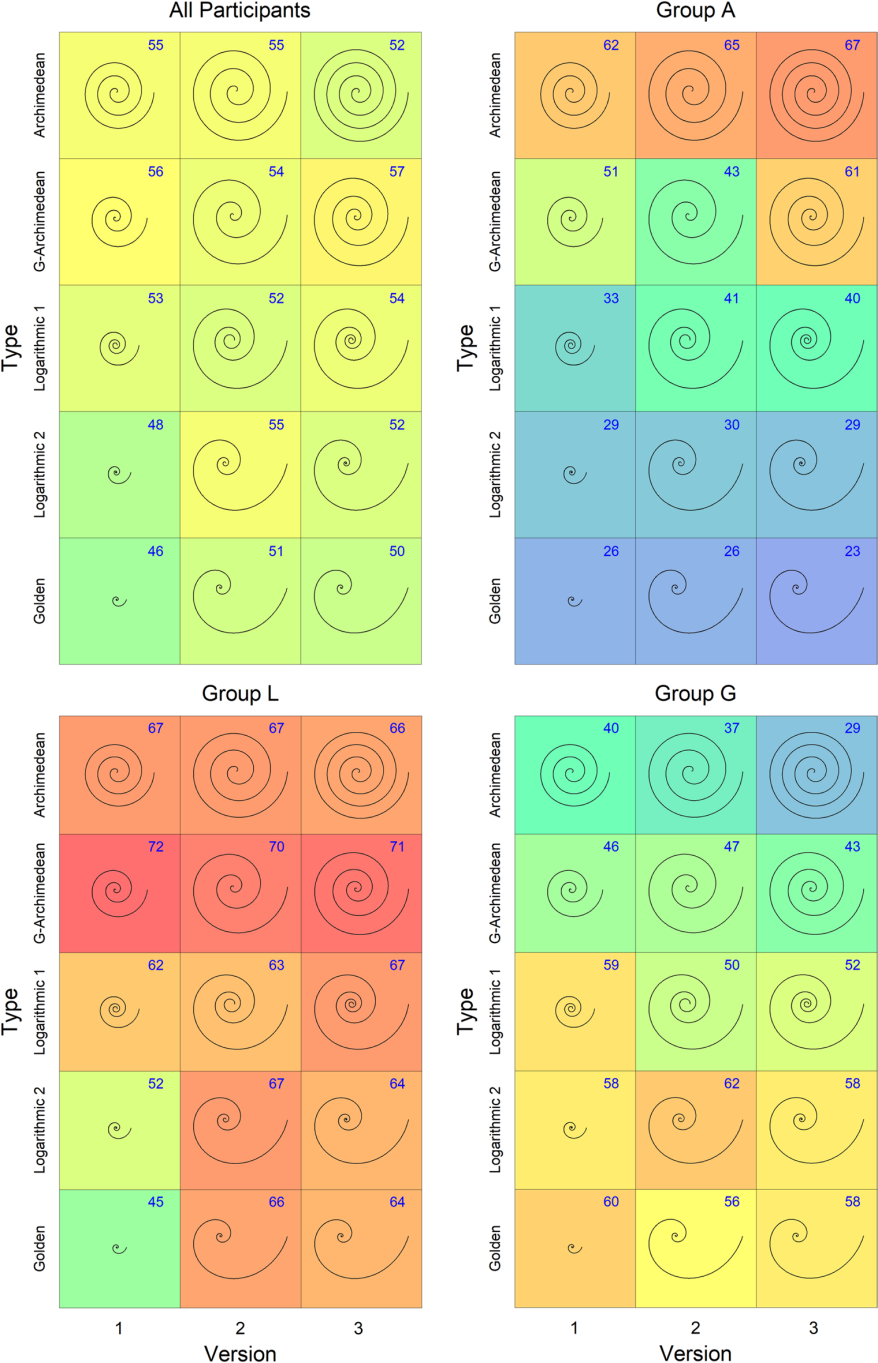
Mean beauty ratings of the spiral stimuli in Experiment 1. The means are shown as a number in the top right-hand corner of each square and as a color of a heat map. The range of the heat map goes from 23 (blue) to 72 (red). The top left panel shows the overall means, whereas the other panels show the means of the three identified groups, respectively.

The overall mean rating was 52.6, and the range of mean ratings across spirals varied from 46 to 57, which is a rather narrow range (see the top left panel in Fig. 2). Therefore, we examined the rater consistency by computing the intraclass correlation (ICC) with the function ‘icc’ from the R Package ‘IRR’^18^. As result, we obtained an ICC(C,118) of 0.591 (two-way, average), which is at the lower end of a moderate consistency^19^. Because the low ICC suggested that our sample included groups varying in preference, we computed a cluster analysis.

#### Cluster analysis

**Fig. 3:**
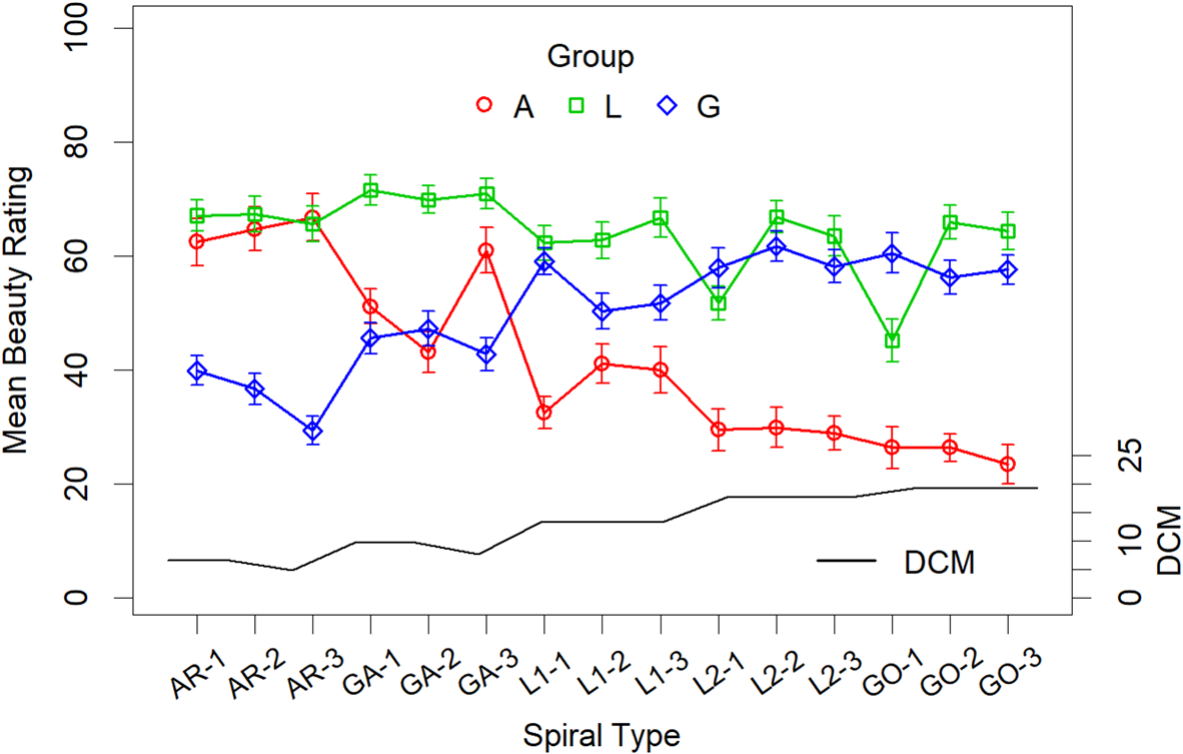
Mean beauty ratings of the spirals by the three groups of participants in Experiment 1. The error bars represent the standard error. The balance scores (DCM, Deviation of the Center of “Mass”) reflect the symmetry of the spirals. Note that the smaller the value, the more symmetrical the spiral (see the text for details).

The function ‘kmeans’ from the R package ‘stats’^20^ found a good solution for three groups (*k* = 3), which explains 33% of the variance. The rater consistency within each group was rather high (Group A: ICC(C,33) = 0.96; Group L: ICC(C,40) = 0.82; Group G: ICC(C,45) = 0.92). The addition of a fourth group only increased the explained variance to 37%. We therefore limited ourselves to three groups and calculated further analyses for each group separately. As can be seen in Figs. 2 and 3, the beauty ratings largely differ between the groups. One group (33 members, 28%), which we call *Group A*, strongly preferred AR spirals to the other types, whereas another group (45 members, 38%), *Group G*, preferred GO and L2 spirals to the other types. Obviously, Group A and Group G had opposite preferences. The last group showed less differentiation in their preferences. However, since their members seemed to slightly prefer spirals with a moderate expansion rate, we will call them *Group L*.

The exact differences and relationships between the groups can perhaps be seen most clearly by examining the line graphs in Fig. 3. For instance, the fact that the values for Group A are markedly decreasing and those for Group G markedly increasing reflects the strong negative correlation between these Groups, *r* = − 0.96, *t*(13) = − 11.7, *p* < 0.001. The correlations between Group L and the other two groups approach significance (with Group A, *r* = 0.509, *p* = 0.053; with Group G, *r* = − 0.487, *p* = 0.065).

When we analyzed how gender is distributed across the groups, we found significant differences, *Χ*^2^(2) = 10.5, *p* = 0.005. The proportion of men was higher in Group A than in the other two groups (Group A: 55%, Group L: 25%, Group G: 23%).

Given the great rating differences between the groups, further overall analyses of the data made little sense. Therefore, we first analyzed the effects of size separately for the three groups, and then the effects of turns.

#### Effects of size

The appropriate data (for Version 1 and Version 2) were subjected to an ANOVA for each group with the within-participant factors *type* (AR, GA, L1, L2, and GO) and *size* (small, and large).

**Group A**. The main effect of *type* was significant, *F*(2.79, 89.3) = 40.9, *p* < 0.001, Generalized Eta Squared (GES) = 0.319, but not that of *size*, *F*(1.00, 32.0) = 0.133, *p* = 0.716, GES = 0.000. The interaction was also not significant, *F*(4.00, 128.0) = 2.30, *p* = 0.062, GES = 0.018. The means for *type* are 63.6, 47.1, 36.8, 29.7, and 26.3, respectively. Bonferroni adjusted *t*-test for *type* revealed significant differences between all combinations except that of GO and L2 type, and of L1 and L2 type. The numerical mean ratings for the individual spirals are also shown in Fig. 2, Group A, Versions 1 and 2.

**Group L**. There were significant main effects of *type*, *F*(4.00, 156) = 7.56, *p* < 0.001, GES 0.078. The corresponding means are 67.2, 70.7, 62.6, 59.3, and 55.6. Bonferroni adjusted *t*-test for *type* revealed significant differences between the mean ratings of the AR and GO type, and between the mean ratings of the GA and the GO, the L1, and the L2 type. The factor *size* was also significant, *F*(1.00, 39.00) = 11.9, *p* < 0.001, GES 0.034. The means are 59.6 and 66.6. However, there was also a significant interaction between the two factors, *F*(3.25, 126.59) = 7.34), *p* < 0.001, GES 0.057. As can be seen in the corresponding panel in Fig. 2, size had a much larger effect for the L2 and GO spirals than for the others. The numerical mean ratings for the individual spirals are also shown in Fig. 2, Group L, Versions 1 and 2.

**Group G**. The main effect of *type* was significant, *F*(3.08, 135.73) = 18.0, *p* < 0.001, GES = 0.144, but not that of *size*, *F*(1.00, 44.00) = 1.29, *p* = 0.263, GES = 0.003. The interaction was also not significant, *F*(3.25, 143.00) = 1.82, *p =* 0.141, GES = 0.013. The means for the types are 38.3, 46.4, 54.7, 59.8, and 58.4. Bonferroni adjusted *t*-test for *type* revealed that the mean rating for the AR spirals differs significantly from those of all other types. This was also the case for the GA. The mean ratings of the GO spirals and those of the other two logarithmic types did not significantly differ from each other. The numerical mean ratings for the individual spirals are also shown in Fig. 2, Group G, Versions 1 and 2.

#### Effect of number of turns

For analyzing the effect of the number of turns the appropriate data (for Version 2 and Version 3) were subjected to an ANOVA for each group with the within-participant factors *type* (AR, GA, L1, L2, and GO) and *turns* (3, and 4).

**Group A**. The main effect of *type* was significant, *F*(2.84, 90.8) = 43.6, *p* < 0.001, GES = 0.350. The type means are 65.7, 52.0, 40.5, 29.4, and 24.9. Bonferroni adjusted *t*-test for type revealed a significant difference between all types, except between L2 and GO. The main effect of *turns* was not significant, F(1.00, 32.0) = 1.85, *p* = 0.184, GES = 0.005. However, there was a significant interaction between the two factors, *F*(4.00,128.0) = 4.39, *p* = 0.002, GES = 0.034. As can be seen in Fig. 2, increasing the number of turns had a positive effect for the first three spiral types, but none or even a negative one for the other two types. The numerical mean ratings for the individual spirals are also shown in Fig. 2, Group A, Versions 2 and 3.

**Group L**. The main effect of *type* was not significant, *F*(3.19, 124.6) = 0.913, *p* < 0.442, GES = 0.012, nor was the effect of *turns* significant, *F*(1.00, 39.0) = 0.061, *p* = 0.806, GES = 0.000, or the interaction between the two factors, *F*(4.00, 156.0) = 0.613, *p* = 0.654, GES = 0.004. The numerical mean ratings for the individual spirals are also shown in Fig. 2, Group L, Versions 2 and 3.

**Group G**. The main effect of *type* was highly significant, *F*(3.26,143.56) = 24.4, *p* < 0.001, GES = 0.201. The mean ratings for type are 33.0, 45.0, 51.0, 60.0, and 57.0. Bonferroni adjusted *t*-test for *type* revealed significant differences between all pairs except that of GA and L1, that of L1 and GO, and that of L2 and GO. The main effect of *turns* (mean for 3 turns is 50.4, and mean for 4 turns is 47.9) fell just short of significance, *F*(1.00, 44.00) = 3.74, *p* = 0.060, GES = 0.004, and the interaction between the two factors was not significant either, *F*(4.00,176.00) = 1.15, *p* < 0.337, GES = 0.008. The numerical mean ratings for the individual spirals are also shown in Fig. 2, Group G, Versions 2 and 3.

#### Correlation

In addition to analyzing the effects of the directly and more categorically varied variables, we also wanted to examine the effects of indirectly co-varied features of the spirals. Of the various candidates, we considered those that seemed potentially relevant: *mean curvature*, *variance of curvature*, *width*,* width-to-height ratio*,* path length*, and *balance* (*symmetry*), where *width* and *width-to-height ratio* were suggested by the reviewers. As measure for balance, we used the DCM (Deviation of the Center of “Mass”), as formalized by Hübner and Fillinger^21^. It should be noted that the balance is usually computed for a whole image. Here, however, we were interested in the balance within each spiral. For Version 2 and Version 3 of the spirals, this balance largely coincides with the balance of the whole image. However, this is not the case for Version 1. Therefore, we used the balance values computed for Version 2 as DCM measures of the corresponding spirals of Version 1. The correlation between this DCM measure and the width-to-height measure is quite high (0.962), while width does not correlate significantly with DCM (− 0.255, *p* = 0.360).

The correlations of these features with the mean ratings of the spirals for each of the three groups can be seen in Table 2. The correlations of the DCM with the mean ratings are also shown graphically in Fig. 3. As can be seen in the Table, for Group L all features correlated with the mean ratings. For the other two groups, however, *variance of curvature* and *width* did not correlate significantly with the mean ratings. Moreover, as could be expected, the signs between the groups were different. In this respect it should be noted that the DCM score decreases with an increasing balance.

**Table 2:** Correlations between the mean beauty ratings and some features of the 15 spirals for each of the three preference groups.

Spiral features	Group A	Group L	Group G
Mean curvature	−0.581*	−0.753**	0.523*
Variance of curvature	−0.484	−0.633*	0.414
Width	0.277	0.749**	0.350
Width-to-Height Ratio	−0.906***	−0.563*	0.826***
Path length	0.842***	0.578*	−0.888***
DCM (Balance)	−0.971***	−0.551*	0.929***

Note. Significance codes *p < 0.05, **p < 0.01, ***p < 0.001.

#### Multiple regression

After considering the correlation of relevant features with the mean ratings for each of the three groups, we wanted to examine how well the ratings can be predicted by these features. For this objective, we computed linear multiple-regression analyses. It turned out that the most successful and at the same time parsimonious model was the one that included *width*, *path length* and balance (DCM). The parameters and *R*^2^values of each Group are provided in Table 3. As can be seen, in addition to *path length*, the *balance* (*symmetry*) played a major role for Group A and Group G, even though in opposite directions.

**Table 3:** Coefficients of multiple-regression analyses. Mean ratings were predicted by relevant features of the spirals.

Predictors	Group A	Group L	Group G
Intercept	67.6***	77.2***	44.4***
Width	−0.974	4.55***	0.919
Path length	0.364	−0.642***	−0.456*
Balance (DCM)	−2.23***	−1.66***	0.876*
*R* ^**2**^	0.957***	0.897***	0.928***

Note. Significance codes: *p < 0.05, **p < 0.01, ***p < 0.001.

The high percentage of explained variance clearly is due to averaging the data. To also analyze the individual data, we applied a linear mixed-effects model (LMM). In this model, individuals were included as a random effect, allowing each participant to have their own intercept. We additionally incorporated the pre-defined groups (A, L, G) as a categorical fixed variable. The specific model used was: `Rating ~ (Width + Path Length + DCM) * Group + (1 | Individual)`. To facilitate interpretation and comparison, the continuous predictors (Width, Path Length, and DCM) were standardized (Z-scored), such that the *β* coefficients represent changes in standard deviations of the outcome variable.

The results of the analysis are summarized in Table 4. As can be seen, the baseline rating (intercept) for the reference Group A was statistically significant. For Group A, Path Length exhibited a small but significant positive effect on ratings, while DCM showed a strong and significant negative effect. Width did not have a significant effect for Group A.

The main effects of Group are represented by the differences in baseline ratings relative to Group A. Both Group L and Group G showed highly significant positive average ratings compared to Group A.

**Table 4:** Results of the linear Mixed-Effects model predicting the ratings in Experiment 1 (see text for details).

Predictors	Rating				
	*β*	*SE*	*t-value*	*df*	*p*
Intercept	−0.451	0.060	−7.51	1756	< 0.001***
Width	−0.075	0.059	−1.28	1756	0.201
Path Length	0.198	0.097	2.04	1756	0.041*
DCM	−0.469	0.076	−6.17	1756	< 0.001***
Group L (vs. Group A)	0.931	0.081	11.5	1756	< 0.001***
Group G (vs. Group A)	0.355	0.079	4.49	1756	< 0.001***
Width × Group L	0.427	0.079	5.38	1756	< 0.001***
Width × Group G	0.146	0.077	1.89	1756	0.059
Path Length × Group L	−0.548	0.131	−4.18	1756	< 0.001***
Path Length × Group G	−0.447	0.128	−3.50	1756	< 0.001***
DCM × Group L	0.118	0.103	1.16	1756	0.248
DCM × Group G	0.653	0.100	6.53	1756	< 0.001***
Random Effects					
σ^2^	0.60				
τ_00 Individual_	0.08				
ICC	0.12				
N _Individual_	118				
Observations	1770				
Marginal *R*^2^/Conditional *R*^2^	0.322/0.401				

Note. Beta (*β*) coefficients are the standardized regression coefficients. They represent the change in the dependent variable in standard deviation units for a one standard deviation increase in the predictor. Important: Group A is the reference category for Group comparisons. *SE* refers to standard error, *df* to degrees of freedom, and *p* to *p*-value. σ² represents the residual variance (unexplained variance within individuals), τ₀₀ the variance of the random intercepts for Individual (between-individual variance). ICC is the Intraclass Correlation Coefficient. Marginal *R*² refers to the variance explained by fixed effects, and Conditional *R*² refers to the variance explained by both fixed and random effects. Significance codes: ***p < 0.001, *p < 0.05.

Considering the interaction effects between the continuous predictors and Group: In contrast to its non-significant effect on the ratings for Group A and Group G, Width had a significant positive effect on the ratings for Group L. Different from its positive effect for Group A, Path Length showed a significant negative effect on the ratings for both Group L and Group G. Finally, in stark contrast to its strong negative effect for Group A, the DCM demonstrated a strong and significant positive effect on the ratings for Group G. For Group L, the effect of the DCM remained negative, and the interaction with DCM was not statistically significant, implying a similar negative effect to that observed in Group A.

Concerning the random effects, we see (ICC) that there is a notable amount of variability (12%) in ratings explained by individual differences, justifying the use of a mixed model.

The Marginal *R*² indicates that 32.2% of the total variance in the ratings can be explained by the fixed effects alone (Path Length, Width, DCM, Group, and all their interactions). When the random intercepts for individuals are also considered, the Conditional *R*² shows that 40.1% of the total variance in the ratings is explained^22^.

Taken together, the LMM results show that, when analyzing the raw, individual observation-level data, a smaller percentage of total variance is explained, compared to our initial multiple regressions, which were performed on aggregated individual means (where within-individual variability was averaged out) for each group. This difference is expected, as the LMM directly accounts for the greater inherent variability present in the original 1,770 individual observations, including both between- and within-individual variance components. Most importantly, the pattern of significant effects of the continuous variables within each group remained consistent with those observed in the separate multiple regressions presented in Table 3.

#### Personality traits (BFI-11)

**Fig. 4:**
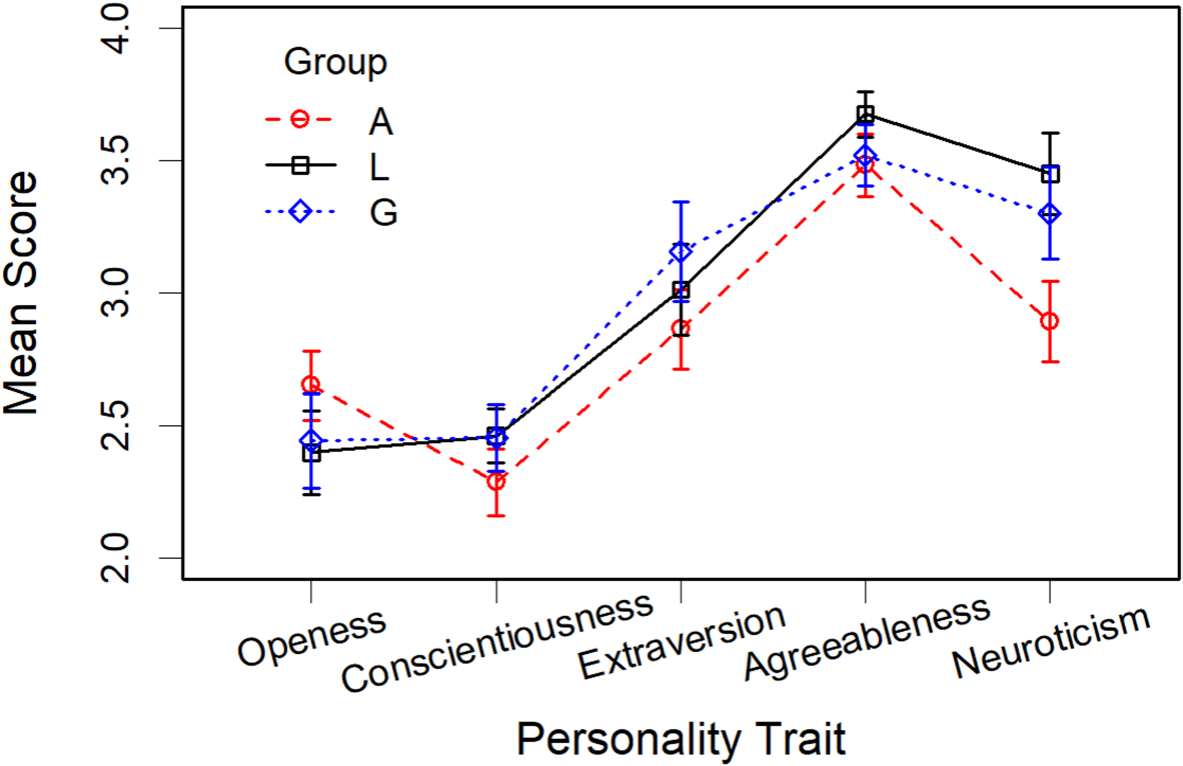
Mean scores of the BFI-11 for each of the three preference groups in Experiment 1. The error bars represent the standard error.

As we identified three groups with different preferences, we wanted to examine to what extent these differences are related to personality traits. For answering this question, we analyzed the results of the BFI-11 for each group. Figure 4 shows the resulting profiles. As can be seen, they are rather similar. The only difference occurred for neuroticism, where Group A had a significantly lower mean score (2.89) than Group L (3.45) and Group G (3.30). However, these differences are confounded with gender. As we have seen, there were more men in Group A than in the other two groups (Group A: 55%, Group L: 25%, Group G: 23%), and it is known that men are usually less neurotic than women^23^. This was also the case in our sample. When we computed an ANOVA with the factors *group* (A, L, G) and *gender* (men, women), then only *gender* was significant, *F*(1, 112) = 6.74, *p* = 0.011, GES = 0.06, but not *group*, *F*(2, 112) = 1.65, *p* = 0.196, GES = 0.03, and not the interaction *F*(2, 112) = 0.937, *p* = 0.395, GES = 0.02. Men had a lower neuroticism score (2.89) than women (3.40).

## Discussion

Our results show that the perceived beauty of spirals largely depends on their type. However, which type is most preferred largely varies from person to person. Interestingly, we were able to identify three different groups of individuals, two of which had completely opposite preferences. It can be said that Group G generally liked the golden spirals but not the Archimedean ones, while Group A was the opposite. The participants in Group L differentiated less between the spiral types. There was a relatively high preference for Archimedean spirals, generalized Archimedean spirals and logarithmic spirals. However, numerically at least, the generalized Archimedean spirals had the highest ratings, regardless of size. Reducing the number of turns also reduced the beauty of the spirals, but only for Group A and mainly for the generalized Archimedean spirals. Thus, Experiment 1 replicates this aspect of the results in our pilot study.

When looking at the spiral types selected for this experiment and the corresponding beauty ratings, it becomes clear that the different preferences could also largely reflect differences in preference regarding the spirals’ expansion or growth. That is, Group A preferred spirals with a linear expansion, which makes the spirals fairly symmetrical and balanced. In contrast, Group G preferred spirals with a rapidly increasing expansion, which makes the spirals strongly asymmetrical and unbalanced. Finally, Group L preferred spirals with a moderately increasing expansion. Since the perceived degree of expansion increases with size and the number of turns, this explains why these characteristics also have a certain influence on the perceived beauty, albeit only to a small extent and only for certain preference groups.

Thus, the concept of expansion seems to be a relatively general property of our spirals that well reflects their differences. However, for a more detailed and formal analysis (multiple regression) neither expansion nor the varied factors (type, size, turns) seem to be appropriate. What was needed was a set of measurable low-level features of the spirals that, in the best case, also represents the concept of expansion. We considered various features such as curvature, path length, balance etc. It turned out that of the various features some are highly correlated. Therefore, we finally decided to use width, path length and balance (symmetry) as predictor variables. With this small number of variables, we were able to explain about 90% of the variance in the mean beauty ratings for each Group.

However, the multiple regressions were calculated separately for each group (A, L, G) using values averaged over the individual data within each group. A linear mixed-effects model (LMM) applied to all raw, individual-level ratings revealed a considerably lower percentage of explained variance. The fixed effects accounting for 32.2% of the total variance (Marginal R²) and the combination of fixed and random effects explaining 40.1% of the total variance (Conditional R²) in the ratings. This disparity is anticipated, as averaging data within individuals inherently reduces within-individual variability, leading to potentially inflated R-squared values. Despite this difference in explained variance magnitudes, it is important to note that the pattern of significant effects for the predictor variables in the LMM mirrored those observed when predicting the mean ratings for each group separately.

The analyses revealed distinct patterns of effects for the continuous predictors across the groups. For participants in Group A, longer path length led to higher ratings, whereas a higher balance (DCM) corresponded to significantly lower ratings. Width, however, had no significant effect for Group A.

Path Length showed a significant negative effect on ratings for both Group L and Group G. Regarding Width, while it demonstrated no significant effect for Group A, it exerted a significant positive influence on ratings for Group L, and no significant effect for Group G. Lastly, in contrast to its strong negative effect for Group A, the DCM had a significant positive effect on ratings for Group G. For Group L, the effect of DCM remained negative, and the interaction with DCM was not statistically significant, suggesting a similar negative influence to that observed in Group A.

Different from our expectation, the differences in preference between the groups do not seem to be substantially related to personality traits, at least not those measured by the BFI-11^17^. There were some indications that the members of Group A were less neurotic. However, since there were significantly more men in this group, who are known to be less neurotic than women^23^ and this was also the case in our experiment, these variables are confounded.

It therefore remains unclear which personality traits, if any, are related to the observed differences in preferences. Although we have identified two key objective features of the spirals - path length and balance - both of which are related to expansion, and whose different aesthetic appreciation largely explains the differences in preference, it remained unclear whether they are also the psychologically effective features. We therefore conducted a second experiment to find an answer to this question.

## Experiment 2A

The results of Experiment 1 indicated that the observed preference differences are related to the path length, width and balance of the spirals. However, it remained uncertain whether these features were also the psychologically effective properties. Participants might have based their decisions on other features as well. For instance, Fechner^24^ already proposed that, besides basic features, associations can also have a strong influence on aesthetic preferences (for a translated version of Fechner, 1866, see^25^). Indeed, depending on the degree of expansion, a spiral can resemble different objects in our environment, which, based on their value to the individual, can affect preferences accordingly.

Therefore, to explore why individuals prefer one type of spiral over another, we not only asked participants to choose between different types of spirals, but also requested them to provide the reasons for their preference. This way, we hoped to obtain information about the individual factors that influence preferences.

### Method

#### Participants

Eighty participants (mean age 28.5 years, SD = 6.62, 55 cisgender women, 25 cisgender men), predominantly students from the University of Konstanz, Germany, were recruited to participate in the experiment. Forty-three of them (17 men), had already participated in Experiment 1. The corresponding online study also included another experiment, which is reported on elsewhere. Participants received a voucher worth EUR 2 for taking part in the study, which lasted around five minutes in total. The experiment was conducted under the same ethical conditions as Experiment 1.

#### Stimuli and procedure

A selection of the spirals used in Experiment 1 served as stimuli. In order to have an approximately even variation of expansion, we used the Archimedean spiral (AR), the logarithmic-1 (L1) spiral, and the golden spiral (GO), each in Version 3, i.e., all spirals had four turns. Their balance score (DCM) is: 4.80, 13.4, and 19.3, respectively.

The three spirals were presented pairwise side by side: (AR, L1), (L1, GO), and (AR, GO). They appeared in black on a white 1000 × 500 pixel rectangle on the screen. The temporal order of the pairs was randomized for each participant, whereas the location of spirals within a pair, i.e., whether it occurred on the left or on the right, was randomized across participants. The task of the participants was first to choose the spiral they prefer by clicking on a button below the corresponding spiral. Then a text input window appeared below the spirals and the participants had to write down why they chose the spiral.

### Results

#### Pair comparisons

**Fig. 5:**
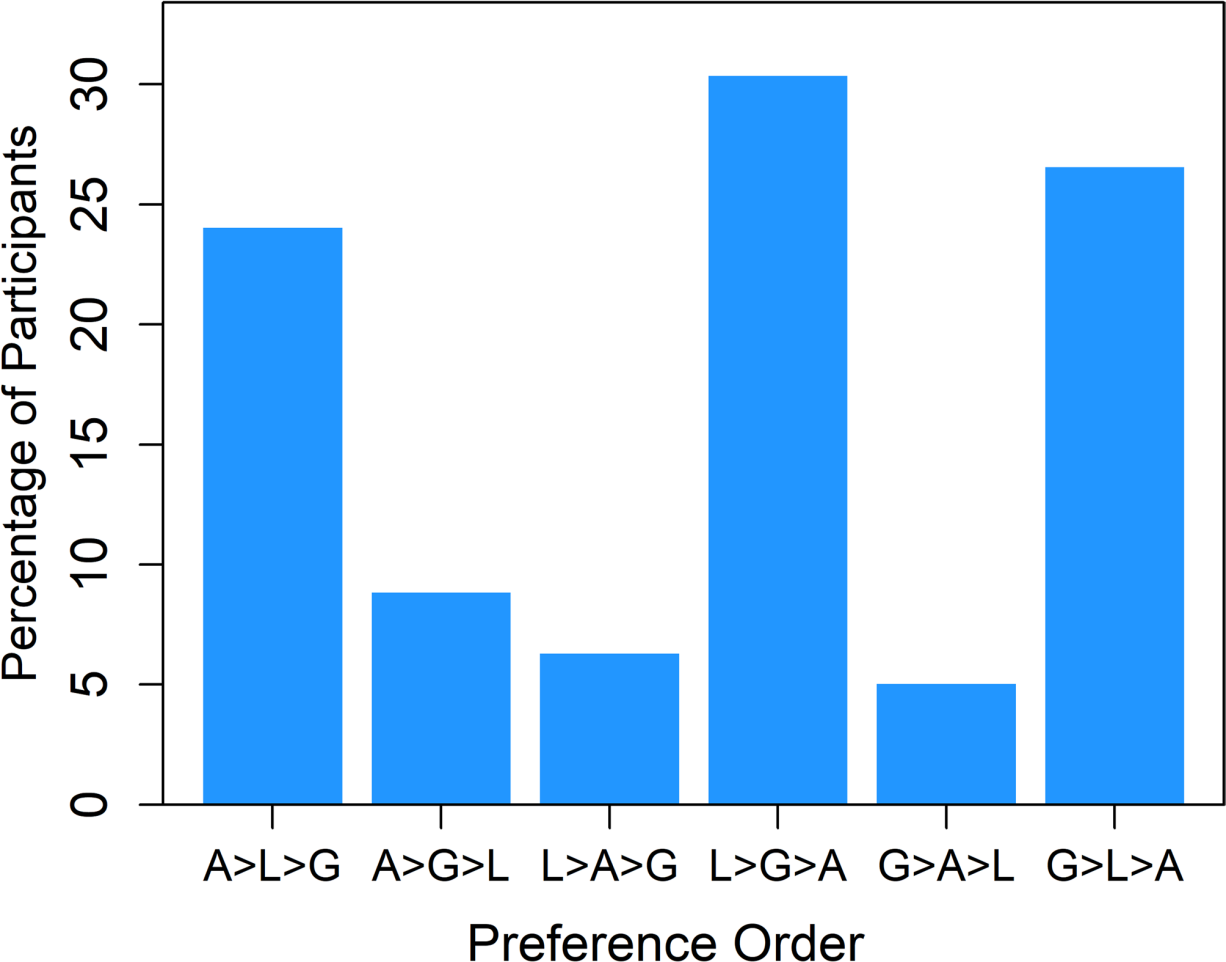
Percentage of participants for each of the six possible preference orders. The label ‘A > L > G’ means that the Archimedean spiral was preferred over the logarithmic spiral, and the latter over the golden one. The meaning of the other labels is analogous.

First, we calculated the order of preference for each participant based on the choices they made. Six different transitive preference orders are possible for the three required choices. As one participant made intransitive choices, this participant’s data was excluded from the analyses. The proportions of the remaining participants, who chose according to a specific preference order can be seen in Fig. 5. They show that 32% (25) of the participants generally preferred the Archimedean spiral, 36% (29) the logarithmic spiral, and 32% (25) the golden spiral. Thus, similar to Experiment 1, there were three preference groups of similar size, which we again refer to as Group A, Group L, and Group G, respectively. As in Experiment 1, the gender ratios varied between the groups. However, while the proportion of men was again highest in Group A (48%; 55% in Experiment 1), the proportions of men in Group L (21%) and Group G (28%) differed from those in Experiment 1 (L: 25%, G: 23%).

As suggested by one reviewer, we also computed preference scores from the pair comparisons. For obtaining overall scores we used the program *BTm* from the R-package *BradleyTerry3*^26^. The resulting scores, which are considered interval scaled, are 0.000, 0.659 and 0.381, for the Archimedean, logarithmic and for the golden spiral, respectively, meaning that the Archimedean spiral was least liked and the logarithmic spiral was most liked. Unfortunately, this method does not work with the data of the preference groups, as one spiral type has always the highest rank for each person. Therefore, we applied the so-called Borda-sum ranking rule^27^ which lead to ordinally scaled scores. In our case with three spiral types and three corresponding pair comparisons, the rule assigns for each participant 2 points to the spiral that was preferred twice, 1 point to the one preferred once and 0 points to the spiral never preferred. Summing up the points across participants and normalizing revealed as preference scores for the three spiral types (Archimedean, logarithmic, golden) for Group A: 0.667, 0.240, 0.093, for Group L: 0.057, 0.667, 0.276, and for Group G: 0.053, 0.280, 0.667. The scores reflect the rank order of the spirals in each group.

#### Reasons

In the next step, we examined the reasons participants stated for preferring the specific spiral within a given pair of spirals. Some participants gave more than one reason. In these cases, each reason was treated separately and independently.

In total, the 79 participants gave 335 reasons for their six choices. The 28 participants who preferred AR to L1 gave, on average, 1.18 reasons, the 51 who preferred L1 to AR gave 1.59 reasons, the 32 who preferred GO to L1 gave 1.34 reasons, the 47 who preferred L1 to GO gave 1.40 reasons, the 29 who preferred AR to GO gave 1.28 reasons, and the 50 who preferred GO to AR gave 1.50 reasons for their choice.

**Fig. 6:**
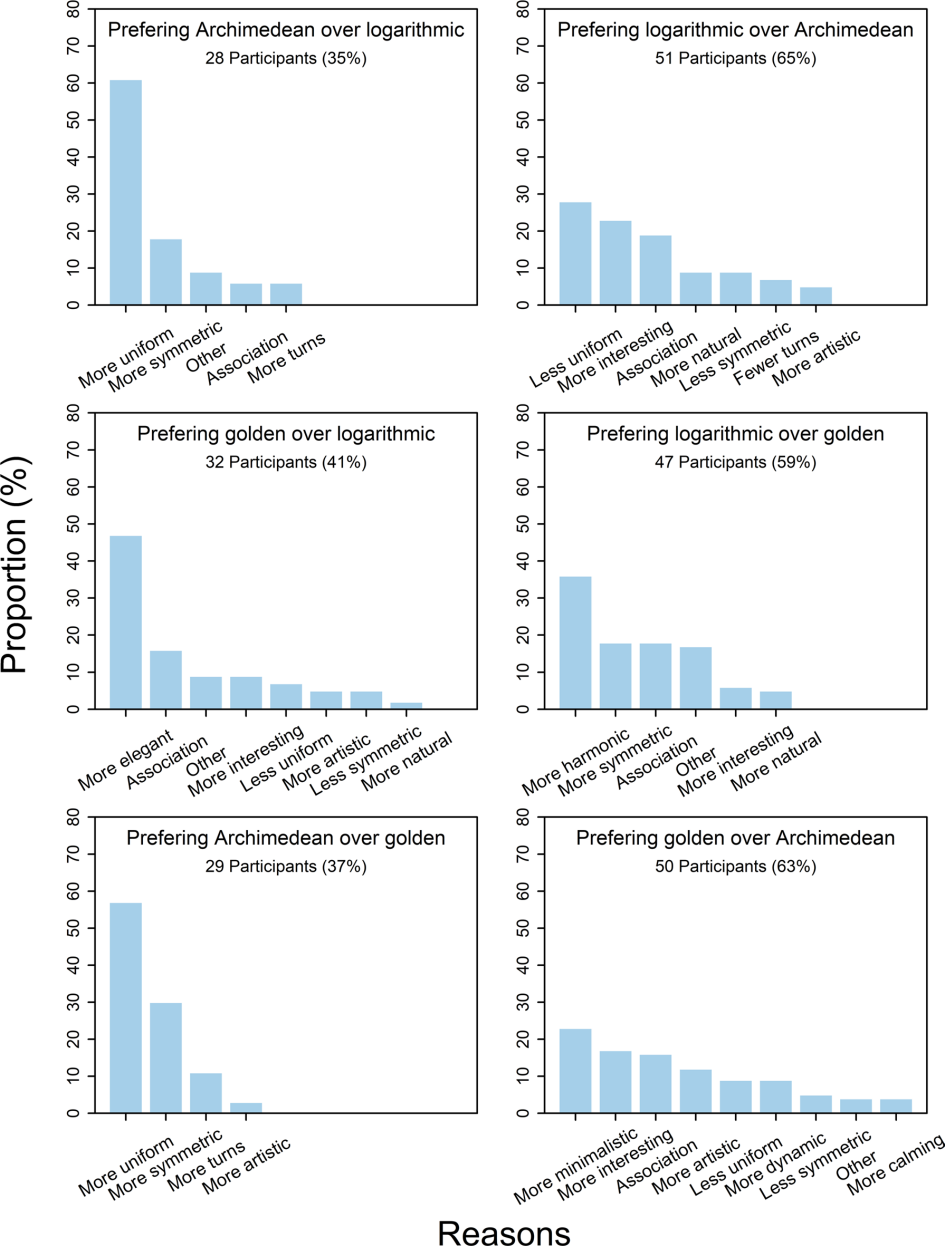
Reasons for the possible choices for each of the three spiral pairs.

In order to examine the frequency of the different reasons given for each decision in view of a particular pair of spirals, they were categorized. The labels of the tick marks in the bar graphs in Fig. 6 indicate which categories occurred in each case. There was obviously the greatest agreement among the participants who preferred the Archimedean spiral. The most common reason given for this choice was the greater uniformity of this spiral, followed by the higher symmetry. Other reasons were rarely mentioned.

For the participants who preferred the logarithmic spiral to the Archimedean spiral, the main reasons were its lower uniformity, that it was more interesting and the associations it evoked, mostly “snail”. When the logarithmic spiral was preferred over the golden spiral, its greater harmony was mentioned most frequently, followed by the greater symmetry and associations.

The lowest level of agreement was found among those who preferred the golden spiral to the Archimedean spiral. The most frequently stated reason was the minimalistic nature of the golden spiral, followed by its greater interestingness and the associations it evoked (often “wave”). There was more agreement among the participants who preferred the golden spiral to the logarithmic spiral. Here, the most frequent reason given was its greater elegance, followed by evoked associations.

## Discussion

Although the three preference groups were categorized using a different method than in Experiment 1, their size was quite similar. This indicates that the preference differences in relation to the spiral types are a reliable phenomenon. The question in this experiment, however, was why the groups differ. While the analyses in Experiment 1 only provided indirect indications of the basic or higher-level features of the spirals in this respect, here, we simply asked our participants to state the reasons for their preferences directly.

There was most agreement among the participants in Group A. Of the reasons given, the greater uniformity of the Archimedean spiral was by far the most frequently mentioned, followed by its greater symmetry. The preference for the Archimedean spiral was therefore mainly based on objective shape features, which at least partially reflect the predictor variables of the multiple regression in Experiment 1. The feature “greater symmetry” corresponds to greater balance and the feature “greater uniformity” corresponds to the constant distance between the turns, which is also reflected by a greater path length compared to the other spiral types (AR: 42, L1: 26, GO: 15).

In contrast to preferring the Archimedean spiral, the preferences for the other two spiral types, the logarithmic spiral and the golden spiral, were frequently based on subjective higher-level characteristics such as interestingness. Moreover, the reasons for preferring one of the other spirals strongly depended on the alternative spiral in a pair. For instance, the reason often given for preferring the golden spiral to the Archimedean spiral was its minimalism and greater interestingness. However, its elegance was very often cited as the reason for preferring it over the logarithmic spiral. Relating these characteristics to the multiple regression predictor variables in Experiment 1 is more difficult. “Elegance”, for example, could perhaps be related as a combination of relatively short path length and asymmetry. Despite the frequent mention of higher-level reasons for choosing one of the non-Archimedean spirals, it should be noted that symmetry (balance) is the only feature whose presence or absence appeared as a reason in all six pair comparisons. This indicates that it generally played some role, and is in line with research showing that individuals differ in their appreciation of symmetry, e.g^28,29^.

Interestingly, associations with the shapes (e.g., snail, wave) were also frequently stated as reasons for favoring the logarithmic or the golden spiral. Since several reasons were often given, it is difficult to say whether associations were the primary or secondary reason. Nevertheless, it was surprising that associations were rarely given for preferring the Archimedean spiral. This raised the question of whether this difference was simply an effect of the type of spiral, i.e., that the Archimedean spiral evokes fewer associations than the logarithmic and golden spirals. Alternatively, the difference could be based on personality differences between the groups, i.e., that members of Group A were simply less able to generate associations. Given the important role of generating associations in creativity^30,^ we hypothesized that at least individuals in Group G might be more creative. This aligns with the observation that several of these participants, considered golden spirals as more artistic and appreciated their asymmetry. Furthermore, Gartus, et al.^29^ have shown that art expertise is linked to a preference for asymmetry. While a detailed investigation of creativity is beyond the scope of this study, we nevertheless wanted to conduct a preliminary experiment to assess the potential of our idea for further research in this direction.

## Experiment 2B

The second part of Experiment 2 aimed to explore potential differences in creativity between the preference groups identified in our previous two experiments. For this objective, we conducted a brief follow-up study with participants from Experiment 2A. Similar to psychometric tests employing divergent thinking tasks^30,^ we required individuals to generate associations to specific prompts. The associations should then be assessed in terms of fluency (quantity) and originality (uniqueness)^30,31^. For the sake of simplicity and because the two corresponding preference groups in Experiment 2 differed the most, we used only the Archimedean spiral and the golden spiral from Experiment 2A as prompts. Our hypothesis was that participants from Group A in Experiment 2A produce fewer and less original associations compared to those from Group G.

### Method

#### Participants

Of the 79 participants in Experiment 2A, 32 also took part in this experiment (mean age 28.8 years, SD = 6.65, 23 women, 9 men). They received no incentive but were promised to receive a description of the objective of the experiment and its results. The experiment lasted only a few minutes, and was conducted under the same ethical conditions as the previous experiments. Of the 32 participants in this experiment, 11 (3 men) were members of Group A, 14 (4 men) of Group L, and 7 (2 men) of Group G from the previous experiment.

#### Stimuli and procedure

The general procedure was similar to that in the previous experiments. Here, however, only the Archimedean spiral and the golden spiral from the previous experiment were used as stimuli and presented in randomized order. For each spiral, the participants were asked to write as many associations as came to mind when looking at the spiral in a text window below the spiral.

### Results

#### Choices

Of the 32 participants 7 (3 men) preferred the Archimedean spiral and 25 (6 men) the golden spiral. The preferences of most participants (84%) were consistent with the previous experiment. Merely five participants altered their choices. Specifically, one participant chose the golden spirals after showing the rank order A > L > G in Experiment 2A. Of the remaining four participants who changed, two shifted their preference to the golden spiral from A > G > L in Experiment 2A, while two shifted to the Archimedean spiral from L > G > A.

#### Associations

Before analyzing the associations, we preprocessed the words produced by the participants to some extent. First, they were transformed to a canonical form by stemming and lemmatization^32^. This means that words are reduced to their basic form so that different inflected forms of a word can be regarded as the same element. For example, “rotate”, “rotating” and “rotated” are reduced to the same base word “rotate”.

Altogether, the participants produced 138 associations. Of these, 73 (52.9%) were to the Archimedean spiral, and 65 (47.1%) to the golden spiral. On average, each participant produced 4.31 associations, 2.28 to the Archimedean spiral, and 2.03 to the golden spiral.

Taking individual preferences into account, the average number of associations was 3.00, 4.43 and 5.00 for the participants in Group A, Group L, and Group G, respectively, in Experiment 2A. Thus, as hypothesized, the participants that preferred the golden spiral produced more associations than those who preferred the Archimedean spiral. In order to test whether Group G produced significantly more associations than Group A, we also made a statistical analysis. In view of the different group sizes and shapes of the distributions, we decided to carry out a permutation test^33^ by conducting 10,000 permutations of the group labels and calculated the difference in means for each permutation. The resulting *p*-value was calculated as the proportion of permutations where the difference in means was greater than or equal to the observed difference. The obtained *p*-value of 0.047 indicates that the difference in the number of associations between Group A and Group G is statistically significant (one-sided). Obviously, the participants in Group L also produced numerically more associations than those in Group A. Although we had no specific hypothesis in this respect, we also computed a permutation test with the data of these groups, which shortly failed to reach significance, *p* = 0.083 (one-sided).

**Fig. 7:**
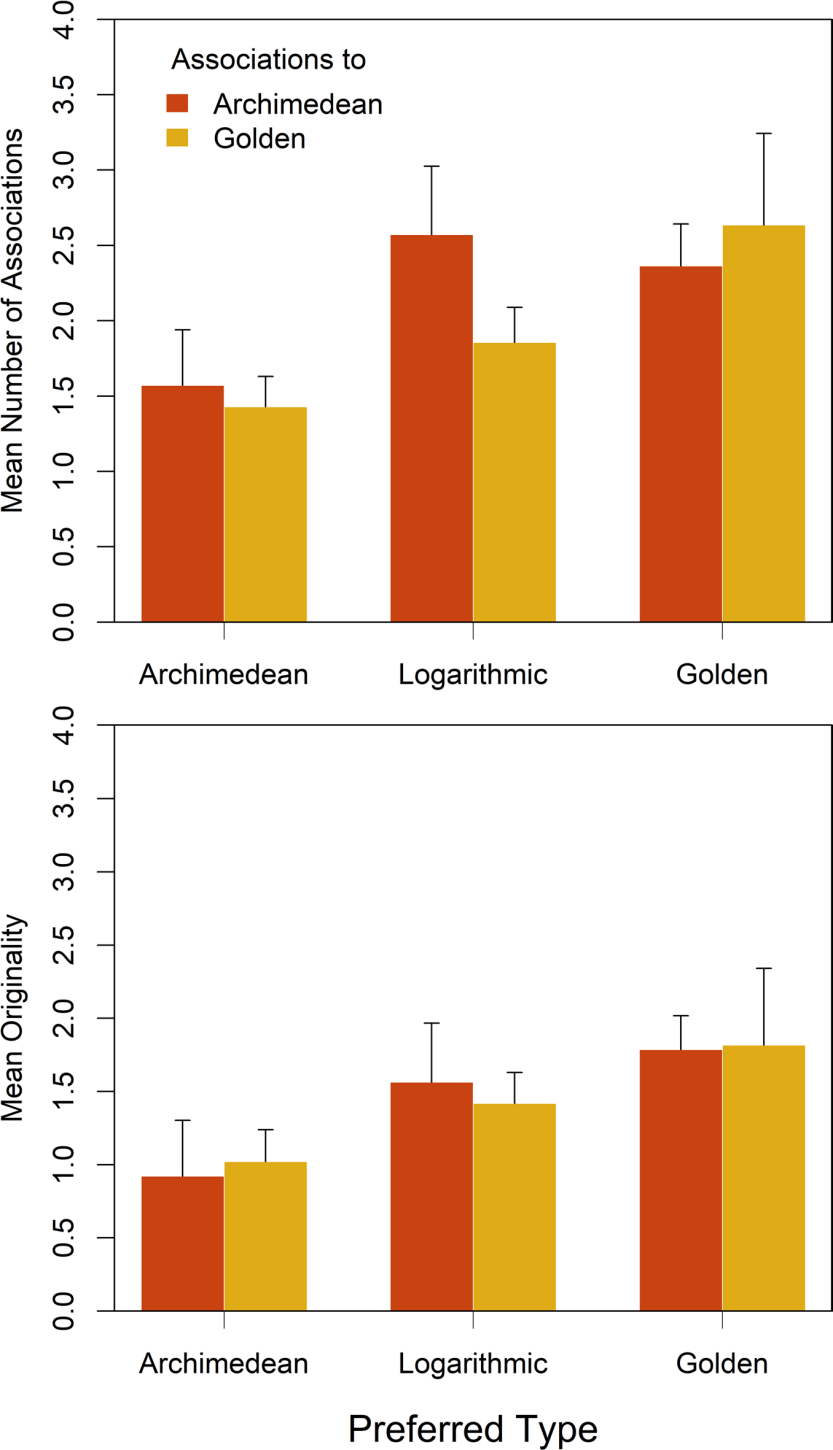
Upper panel: The mean number of associations produced by each participant in Experiment 2B depending on the spiral type and spiral preference. The error bars represent the standard error. Lower panel: The mean originality of the associations.

If, in addition to the preference, the two spiral types that served as prompts are also taken into account, then the results are as shown in the upper panel of Fig. 7. As can be seen, Group G produced numerically more associations to both spiral types, compared to Group A.

The number of associations that someone produces does not in itself say much about their creativity. The associations could all be obvious. It is therefore instructive to also consider the originality of the associations. This can prevent someone who produces three trivial associations from being judged as more creative than someone who produces only two but very original associations. In our case, we simply took into account the frequency of the individual associations in order to assess their originality, i.e., how often an association was stated for a given spiral. For calculating the frequency of the associations, semantically similar words such as “Schneckenhaus” (snail shell) and “Schnecke” (snail) were considered as the same association. The enumeration revealed “snail” (16.4%), “uniformity” (6.85%) and “labyrinth” (5.48%) as the three most frequent associations for the Archimedean spiral. For the golden spiral, “wave” (7.69%), “snail” (6.15%) and “flowing” (6.15%) were the most frequent associations.

The originality of an association was then defined by 1/*n*, where *n* is its frequency. Thus, a unique association had a value of 1, while an association stated *n* times received a value of 1/*n*. The sum of all originality values of all associations generated by a participant was taken as his or her originality score. Consequently, participants who produced few but original associations could have a higher score than those who produced more but less original associations. The mean originality for each of the different conditions is shown in the lower panel of Fig. 7.

To test whether Group A and Group G also differed in originality, we subjected the corresponding scores to a (one-sided) *t*-test, which revealed a significant difference (1.94 versus 3.60), *t*(15.9) = 1.87, *p* = 0.040, *d* = 0.824.

## Discussion

Although not all participants from the previous experiment could be recruited to take part in this experiment, the results provide evidence for our hypothesis that the preference for certain spiral types is related to creativity. Specifically, individuals who prefer the golden spiral to the Archimedean spiral appear to be more creative than those with the opposite preference, at least in terms of the number and originality of associations generated.

It must be mentioned that originality, as we measured it, usually correlates highly with fluency, which is also the case in our experiment (*r* = 0.914). Unfortunately, it is not possible to say whether this correlation is purely artifactual or a mixture of artifactual and actual correlation^34^. In any case, it shows that a high level of creativity was not based solely on the production of many associations, regardless of their frequency of occurrence.

## General discussion

Given the wide variety of spiral types, this study aimed to investigate the properties that contribute to their beauty. In the first experiment, we explored the aesthetic appreciation of Archimedean, logarithmic, and golden spirals. In addition, we also varied their size and number of turns. Since we found individual differences in aesthetic ratings in a small pilot study with the same spiral types as in the present study, we expected to find similar differences in our Experiment 1, which was indeed the case. Interestingly, three distinct groups could be identified. Group A preferred Archimedean spirals but not golden spirals, while the opposite was true for Group G. Participants in Group L showed some preference for logarithmic spirals and similar ones, but generally differentiated less between spiral types. However, they rated smaller spirals as less beautiful compared to the other groups. Reducing the number of turns decreased the perceived beauty of spirals for Group A, particularly for generalized Archimedean spirals.

The differences in preferences between groups did not appear to be significantly related to personality traits, at least those measured by the BFI-11^17^. There was some evidence suggesting that members of Group A were less neurotic. However, since this group contained significantly more men, who are on average less neurotic than women, this represents a confounding factor. Consequently, it remains unclear whether and which personality traits are associated with the observed differences in preferences.

Multiple regression and LMM analyses suggest that the aesthetic evaluation of three basic features of spirals – width, path length and balance (symmetry)- all related to expansion, can more or less explain the observed preference differences. However, it remained unclear whether these features were also the psychologically effective ones. Therefore, to investigate this issue, we conducted Experiment 2A, where participants chose between an Archimedean, a logarithmic, and a golden spiral presented in pairs. After each choice, they had to state the reason for their preference. Although we cannot be completely sure that the reasons given were actually decisive for the choice, their direct influence was probably more likely than that of the features we used in the regression.

The analysis revealed that the preference for the Archimedean spiral was mainly based on objective shape features that partially reflected the predictor variables from Experiment 1. The most frequent reasons given were “greater symmetry”, corresponding to greater balance, and “greater uniformity”, corresponding to the constant distance, which is also reflected by a greater path length. In contrast, preferences for the other two spiral types, the logarithmic and golden spirals, were often based on subjective higher-level features. Moreover, the reasons for choosing these two spirals depended strongly on the alternative spiral type in a given pair. A possible explanation for this result could be that the golden spiral and the logarithmic spiral are subjectively more similar to each other than to the Archimedean spiral, so that the participants had to focus on different distinguishing features in each case.

In addition to basic and subjective higher-level shape features, associations with the shapes (e.g., snail, wave) were frequently given as reasons for preferring the logarithmic and golden spirals. Since multiple reasons were often provided, it is difficult to determine whether associations were the primary or secondary reason. Nonetheless, the fact that associations were frequently given for the logarithmic and golden spirals, but rarely for the Archimedean spiral, raised the question of whether this difference is simply an effect of the spiral type or indicates personality differences among participants.

As associations play a crucial role in creativity^30^ we hypothesized that participants who prefer these spiral types, especially the golden spiral, might be more creative. This is consistent with the observation that several participants, especially those who preferred the golden spiral, considered it to be more artistic and appreciated its asymmetry. It is also in line with previous research showing that art experts prefer asymmetry^29^.

The results of a follow-up experiment (Experiment 2B), in which a subset of participants from Experiment 2A generated associations to an Archimedean and a golden spiral, support the hypothesis that preference for certain spiral types is related to creativity. As expected, the participants who preferred the golden spiral to the Archimedean spiral in Experiment 2A not only produced more but also more original associations. This supports the idea that these participants were more creative.

Taken together, our findings indicate that spirals have a great potential for investigating aesthetic phenomena. They expand the study by Hübner^5^ which showed that the golden spiral is widely preferred to the Fibonacci spiral, and that this is due to subtle variations in a basic feature, in this case *curvature*. Hübner’s^5^ result supports Fechner’s hypothesis that there are basic features that evoke universal preferences. But not all features are of this kind. If this were the case, there would be little variation in areas such as design and fashion. The variation commonly observed in such fields is a consequence of higher-level features and the associations they evoke^24^ that can be interpreted and aesthetically evaluated in different ways, and which in turn depend on experience, knowledge, attitudes, etc^35^. These higher-level features and corresponding associations are also what lead to individual preferences. The present study provides illustrative examples for such individuality. However, our results obtained with the different spiral types also demonstrate that individual differences are not necessarily arbitrary but rather manifest within more or less clearly defined groups with similar preferences. Without such phenomena, the concept of group-specific design or fashion would be largely ineffective^36^. One factor identified in the present study that can define groups is creativity. Individuals who are fluent and original in generating associations may show different preferences than those with lower levels of creativity.

### Limitations and further direction

Although our study produced interesting and promising results, it also has some limitations. One unresolved issue is the confounding of preference with gender. Accordingly, further data are needed to reliably determine whether or not men tend to prefer Archimedean spirals to golden spirals.

As far as the hypothesized relations between preference, associations, and creativity are concerned, our data are only a first step in this interesting direction. We considered the preference for spirals and also assessed the creativity of the participants based on the frequency and originality of their associations to spirals. Consequently, these two variables are confounded. In further studies, associations with other objects should therefore also be requested and evaluated. If our hypothesis is valid, then they should lead to similar relations and conclusions as in the present study.

Finally, as not all participants in Experiment 2A could be persuaded to also take part in Experiment 2B, the latter experiment is slightly underpowered. Studies with greater statistical power might therefore find that people who prefer logarithmic spirals are also more creative than those who consider Archimedean spirals more beautiful, but less creative than those preferring the golden spiral. Experiment 2B included the participants from Experiment 2A who agreed to also participate in Experiment 2B. This type of self-selection could have led to a certain bias in the results.

### Public significance statement

By examining the beauty of different types of spirals, we uncovered distinct aesthetic preferences among individuals. Our findings reveal that these preferences fall into clear groups, each with a unique taste. Furthermore, our data suggest that a preference for certain spiral types, particularly the golden spiral, may be associated with higher creativity. This insight opens new avenues for understanding how aesthetic preferences can reflect deeper cognitive and creative processes. Overall, our study highlights the potential of spirals as a tool for exploring various aesthetic phenomena, contributing to a broader understanding of how we perceive and appreciate beauty.

## Data Availability

The datasets of this study are available in the OSF repository: https://osf.io/5srax/?view_only=908f86afd3a04342914e5272cfb5207d.
